# S100A10 and its binding partners in depression and antidepressant actions

**DOI:** 10.3389/fnmol.2022.953066

**Published:** 2022-08-15

**Authors:** Michelle X. Chen, Yong-Seok Oh, Yong Kim

**Affiliations:** ^1^University of Iowa Medical Scientist Training Program, Carver College of Medicine, University of Iowa, Iowa, IA, United States; ^2^Department of Brain Sciences, Daegu-Gyeongbuk Institute of Science and Technology (DGIST), Daegu, South Korea; ^3^Department of Neurosurgery, Robert Wood Johnson Medical School, Rutgers University, Piscataway, NJ, United States; ^4^Brain Health Institute, Rutgers University, Piscataway, NJ, United States

**Keywords:** depression, antidepressants, serotonin receptors, Smarca3, Ahnak, mGluR5, annexin A2, Supt6h

## Abstract

S100A10 (p11) is an emerging player in the neurobiology of depression and antidepressant actions. p11 was initially thought to be a modulator of serotonin receptor (5-HTR) trafficking and serotonergic transmission, though newly identified binding partners of p11 and neurobiological studies of these proteins have shed light on multifunctional roles for p11 in the regulation of glutamatergic transmission, calcium signaling and nuclear events related to chromatin remodeling, histone modification, and gene transcription. This review article focuses on direct binding partners of p11 in the brain including 5-HTRs, mGluR5, annexin A2, Ahnak, Smarca3, and Supt6h, as well as their roles in neuronal function, particularly in the context of depressive-like behavior as well as behavioral effects of antidepressant drug treatments in mice. In addition, we discuss neurobiological insights from recently uncovered p11 pathways in multiple types of neurons and non-neuronal cells and cast major remaining questions for future studies.

## Introduction

As a leading cause of disability, major depressive disorder (MDD) affects an estimated 8.5% of adults in the United States and 5% of adults worldwide (Ettman et al., 2020; GBD 2019 Mental Disorders Collaborators, 2022). However, these numbers vary or soar in populations affected by traumatic or life-disrupting events such as natural disasters (Li et al., 2019), epidemics (Ettman et al., 2020; COVID-19 Mental Disorders Collaborators, 2021), military attacks, or civil wars (Naja et al., 2016; Kovess-Masfety et al., 2021).

The etiology of MDD is not well understood, and many genetic and environmental risk factors may contribute to MDD (Wong and Licinio, 2001; Otte et al., 2016). Classic antidepressants including selective serotonin reuptake inhibitors (SSRIs) can take 4–6 weeks to become effective (Otte et al., 2016; Harmer et al., 2017). Additionally, more than one-third of patients have treatment-resistant depression (TRD; Little, 2009; Zhdanava et al., 2021). Recently, a nasal spray formulation of a low dose of ketamine was FDA approved as a fast-acting antidepressant for TRD (Kim et al., 2019). However, the usage of ketamine is highly limited by its psychedelic side effect (Short et al., 2018) and abuse potential for recreational purposes (Sassano-Higgins et al., 2016). Thus, studies of the pathophysiology of MDD and antidepressant actions are crucial to finding innovative targets for this burdensome disorder.

S100A10 (also called p11), a member of the S100 protein family (Donato et al., 2013), levels are reduced in postmortem brains of patients with depression (Svenningsson et al., 2006; Alexander et al., 2010). p11 knockout (KO) mice display depressive- or anxiety-like behaviors, such as increased thigmotaxis in the open field test (OFT) and increased immobility during the tail suspension test (TST). Conversely, p11 overexpression mice display antidepressive-like behaviors, which are behaviors that mimic those induced by antidepressant administration, such as decreased immobility in the TST and forced swim test (FST) and increased sucrose consumption in sucrose preference test (SPT; Svenningsson et al., 2006; Alexander et al., 2010). Antidepressant treatment and electroconvulsive therapy both increase p11 levels in mice (Svenningsson et al., 2006; Oh et al., 2013) *via* epigenetic changes of DNA methylation in the p11 promoter region (Melas et al., 2012; Neyazi et al., 2018) and AP-1 complex-mediated transcriptional regulation (Chottekalapanda et al., 2020). Also, behavioral and neurogenic effects of SSRIs were abolished in p11 KO mice (Svenningsson et al., 2006; Egeland et al., 2010; Warner-Schmidt et al., 2010). These findings initiated a new research avenue into molecular and cellular p11 pathways underlying depressive-like behavior and antidepressant actions (Svenningsson et al., 2013).

Although several review articles describing p11 and its function are available (Svenningsson and Greengard, 2007; Svenningsson et al., 2013; Seo and Svenningsson, 2020), we will discuss recent cell-type-specific studies with a focus on direct interactors of p11 to illuminate the updated view of p11 pathways. Notably, in most studies reviewed in this article, behavioral tests such as FST, TST, novelty-suppressed feeding (NSF), OFT, SPT, elevated plus maze, splash test, and/or cookie test were used to assess behavioral phenotypes. Although translating such behavioral outcomes to human conditions of depression or antidepressant effects is highly limited, we adopt the terminologies of “depressive-like” and “antidepressive-like” to describe the behavioral phenotypes measured in these simple and quantifiable assays. These terminologies are helpful for reviewing studies of molecular and neurobiological mechanisms of p11 and its binding partners.

## P11/5-HTR Pathways in Depressive-Like Behavior and in Antidepressant Actions

**Figure 1:**
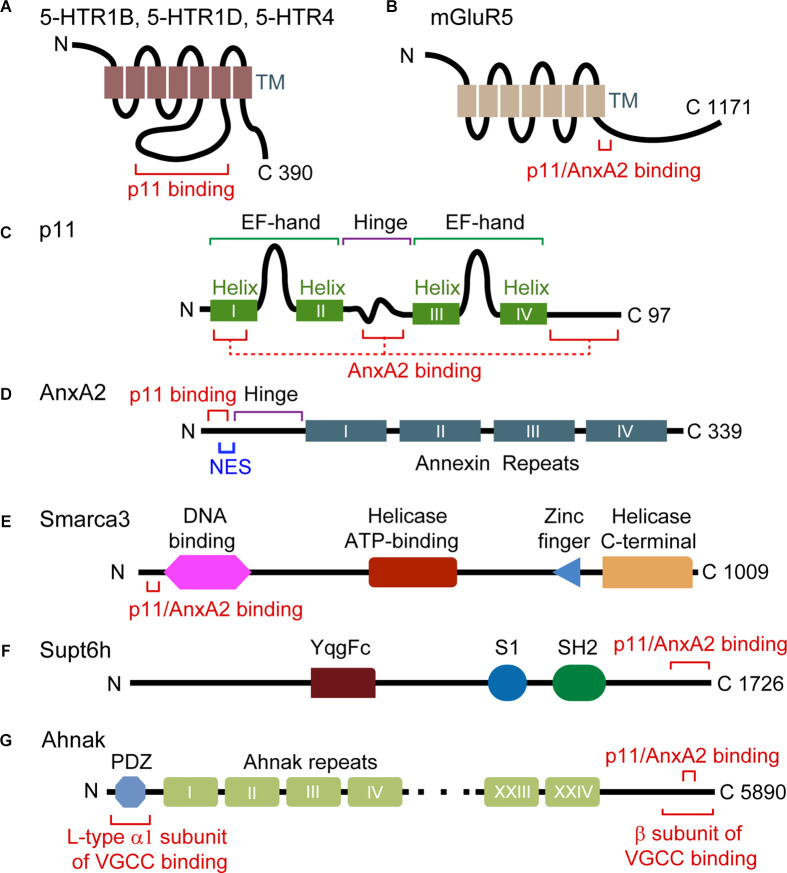
Domain structure and interaction motifs in p11 and p11 binding partners. **(A,B)** 5-HTRs and mGluR5 are G protein-coupled receptors with seven transmembrane (TM) domains. The third intracellular loop is the binding region of p11 **(A)**, and a p11/AnxA2-binding motif exists in the C-terminal tail of mGluR5 (amino acids 836–844) **(B)**. **(C)** p11 protein is composed of two non-calcium-binding EF-hand motifs and four α helices. Helix I, the hinge and C-terminal regions of p11 participate in the interaction with AnxA2. **(D)** AnxA2 consists of a p11-binding motif (amino acids 2–12) and a nuclear export signal (NES) motif in N-terminal region, and four annexin repeat domains. **(E)** Smarca3 is composed of the DNA-binding, helicase ATP-binding, RING-type zinc finger, and helicase C-terminal domains. The p11/AnxA2-binding motif is located in the N terminus region (amino acids 34–39). **(F)** Supt6h is composed of YqgFc, S1, and SH2 domains. A p11/AnxA2-binding motif resides in the C-terminal region (amino acids 1650–1726). **(G)** Ahnak consists of a PDZ domain in the N terminal region and a central region consisting of 128 amino-acid units, repeated 26 times. The p11/AnxA2-binding motif resides in the C-terminal region (amino acids 5663–5668).

**Figure 2:**
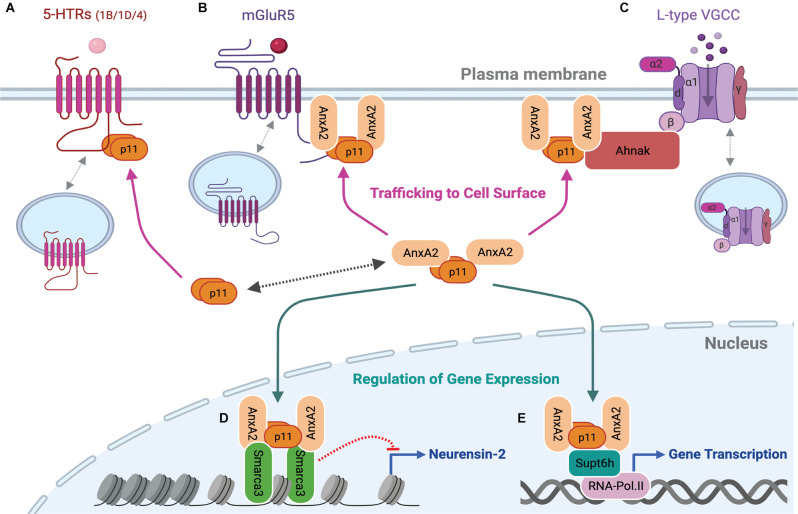
p11 as a functional modulator of its binding partners at the plasma membrane and in the nucleus. p11 dimer or p11/AnxA2 heterotetrameric complex regulates serotonin, glutamate, or calcium signaling by increasing levels of 5-HTRs **(A)**, mGluR5 **(B)** or L-type VGCCs **(C)** at the plasma membrane. The heterotetrameric p11/AnxA2 complex also binds to Smarca3 **(D)** or Supt6h **(E)** and regulates Smarca3- or Supt6h-mediated chromatin remodeling, epigenetic modifications, and gene transcription. These cell surface and nuclear actions underlie depressive or antidepressant-like behavioral effects of genetic modulations of p11 and its binding partners. This figure was created with BioRender.com.

Svenningsson et al. (2006) looked for binding partners of the 3rd intracellular domain of serotonin (5-hydroxytryptamine, 5-HT) receptor 1B (5-HTR1B) in a yeast two-hybrid assay and found p11 (Figure 1A). Subsequent assays with 14 members from the S100 family and 12 subtypes of 5-HTRs confirmed selective binding between p11 and 5-HTR1B, 5-HTR4, and 5-HTR1D (Warner-Schmidt et al., 2009). P11 was required for 5-HTR1B and 5-HTR4 cell surface localization as well as for the behavioral effects of 5-HTR1B or 5-HTR4 agonists (Svenningsson et al., 2006; Warner-Schmidt et al., 2009; Figure 2A).

5-HTR1B, 1D, and 4 are highly implicated in depressive-like behavior and in antidepressant actions (Lowther et al., 1997; Tiger et al., 2018; Murphy et al., 2021). P11 containing neurons co-express these serotonin receptors in the cortex, hippocampus, and caudate-putamen (Egeland et al., 2011; Schmidt et al., 2012). However, mice with constitutive or conditional deletions of 5-HTR1B not only are less anxious but also show phenotypes mimicking antidepressant-induced behaviors in the FST, TST, and SPT (Jones and Lucki, 2005; Bechtholt et al., 2008; Nautiyal et al., 2016). Selective deletion of 5-HTR1Bs from serotonergic neurons in mice was found to increase extracellular 5-HT levels in response to an SSRI in the hippocampus (Nautiyal et al., 2016). Thus, the involvement of p11–5-HTR1B in regulating depressive-like behavior and antidepressant action might be cell type-specific.

p11 is highly expressed in cholinergic interneurons (CINs) in the nucleus accumbens (NAc; Warner-Schmidt et al., 2012), which is a critical brain region controlling reward responses (Nestler et al., 2002). While p11 reduction in CINs in the NAc using an AAV-gene transfer technique to express siRNA targeting p11 or transgenic approaches [ChAT-Cre line bred with mice harboring flanked loxP sites for p11 gene (floxed p11)] causes depressive-like behavior in the TST, FST, and SPT, restoration of p11 levels in CINs in the NAc in the constitutive p11 KO mice sufficiently reversed this depressive-like behavior (Alexander et al., 2010; Warner-Schmidt et al., 2012). The depressive-like phenotype is recapitulated by silencing neuronal transmission in CINs by ChAT-Cre-dependent and AAV-mediated production of the tethered peptide toxin antagonists of Cav2.1 and Cav2.2 calcium channels (Warner-Schmidt et al., 2012). Presynaptic 5-HTR1B in CINs in the NAc are involved in inhibiting acetylcholine release in a p11-dependent manner, and CINs-specific deletion of 5-HTR1B (ChAT-Cre line crossed with floxed 5-HTR1B) induces an anhedonia-like phenotype in the SPT, cookie test, and social approach test (Virk et al., 2016).

p11 deletion in CINs also changes the intrinsic membrane property of CINs by reducing the gene expression of hyperpolarization-activated cyclic nucleotide-gated channel 2 (HCN2; Cheng et al., 2019). The expression of HCN2 and tonic firing of CINs in the NAc are reduced in chronic stress mouse models. Overexpression of HCN2 channels in CINs enhances cell activity and is sufficient to rescue depressive-like phenotypes in CIN-specific p11 KO mice (ChAT-Cre line crossed with floxed p11 line). Notably, p11 regulation of 5-HTR1B function and HCN2 gene expression is cell-type specific because 5-HT1B and HCN2 are not involved in p11-mediated pathways in other cell types including parvalbumin (PV)-expressing interneurons (Medrihan et al., 2020; Sagi et al., 2020). A study suggests the interaction between p11 and 5-HTR1B receptor in cholecystokinin (CCK)-expressing interneurons in the ventral hippocampus might be relevant for behavioral effects of acute administration of antidepressants measured in the TST and FST (Medrihan et al., 2017).

Chronic SSRI administration in mice led to increased 5-HTR4 expression in corticostriatal neurons expressing p11, located in layer 5 of the cerebral cortex (Schmidt et al., 2012). However, instead of 5-HTR4, 5-HTR2 appears to underlie SSRI-mediated changes in the excitatory 5-HT responses in layer 5a p11-expressing neurons (Sargin et al., 2020). In the absence of 5-HTR4, behavioral effects of chronic fluoxetine were still seen in corticosterone-treated mice, such as increased time in the center during the OFT and decreased latency to feeding during the NSF (Amigo et al., 2021). Constitutive deletion of 5-HTR4 does not affect depressive-like behavior (Nautiyal and Hen, 2017). However, deletion of 5-HTR4 specifically from excitatory neurons of the hippocampus results in robust antidepressive-like behaviors and an elevation in baseline anxiety (Karayol et al., 2021), measured by the TST, FST, sucrose splash test, OFT, elevated plus maze, and NSF. These updated studies together with the somewhat different phenotypes found in 5-HTR1B and 5-HTR4 KO mice compared to the phenotype of p11 KO mice indicate the contribution of additional p11 pathways in regulating depressive-like behavior or antidepressant actions.

## p11/AnxA2/mGluR5 Pathways in Depressive-Like Behavior and in Fast-Acting Antidepressant Actions

Previous studies of antagonists acting on metabotropic glutamate receptor 5 (mGluR5) or mGluR2/3 indicate their role in phenotypes that mimic antidepressant-induced behaviors in mice (Belozertseva et al., 2007; Chaki et al., 2013; Goeldner et al., 2013; Palucha-Poniewiera et al., 2013). Interestingly, the antidepressant-like effect of a selective antagonist of mGluR5 but not of an mGluR2/3 antagonist, seen in wild-type (WT) mice was abolished in constitutive p11 KO mice in FST and NSF (Lee et al., 2015). mGluR5 and p11 mutually facilitate their accumulation at the plasma membrane. This specific role for p11 in cell surface localization of mGluR5 is due to the direct interaction of the p11/annexin A2 (AnxA2) heterotetrameric complex with mGluR5 (Lee et al., 2015; Figures 1B, 2B), and p11 interaction with AnxA2 is previously well characterized (Kwon et al., 2005; Rescher and Gerke, 2008; Hedhli et al., 2012; Figures 1C,D).

The imbalance of glutamate and γ-aminobutyric acid (GABA) systems plays a central role in the pathophysiology of MDD (Sanacora et al., 2012; Duman et al., 2019; Sarawagi et al., 2021). mGluR5 is expressed both in glutamatergic neurons and inhibitory interneurons (van Hooft et al., 2000; Lopez-Bendito et al., 2002; Sun et al., 2009), in which stimulation of Gq-coupled mGluR5 positively regulates neuronal activity (De Blasi et al., 2001). mGluR5 receptor deletion selectively in GABAergic interneurons (GAD-Cre or PV-Cre line crossed with floxed mGluR5) renders an antidepressive-like behavioral phenotype, while its deletion in forebrain glutamatergic neurons (EMX-Cre line crossed with floxed mGluR5) results in a depressive-like phenotype in the TST, FST, and SPT (Lee et al., 2015). This suggests an overriding role of chronic inhibition of PV interneurons in antidepressant-like behavioral outcomes observed in mGluR5 null mice (Li et al., 2006). Consistent with this notion, pharmacological inhibition of mGluR5, and thereby disinhibition of PV neuron-mediated enhanced glutamatergic neuronal activity, underlies a fast-antidepressant-like activity of an mGluR5 antagonist in rodents (Lee et al., 2015).

The critical role of the glutamate system in antidepressant actions is mirrored by ketamine, an inhibitor of NMDA receptor. One mechanism underlying the antidepressant actions of ketamine is an increase in glutamatergic activity *via* transient inhibition of inhibitory interneurons (Duman et al., 2019; Gerhard et al., 2020; Fogaca et al., 2021). Chronic unpredictable mild stress decreases hippocampal p11 level in rats, which is significantly recovered to control levels 72 h after ketamine administration (Sun et al., 2016). Ketamine-induced p11 restoration is dependent on TrkB activity, which is consistent with the role of BDNF in p11 induction (Warner-Schmidt et al., 2010; Chottekalapanda et al., 2020). Lentiviral vector-mediated p11 knockdown in the hippocampus causes depressive-like behavior in FST and SPT and blocks antidepressant-like effects of ketamine in a chronic stress paradigm, suggesting a role for p11 in ketamine-mediated antidepressant effects (Sun et al., 2016). These results suggest that p11 pathways are involved in fast-acting antidepressant actions in rodent models.

## p11/AnxA2/Smarca3 Pathways in Antidepressant Actions of SSRIs

When p11 binding partners were searched for using a heterologous expression system of Hek293 cells, Smarca3 and Supt6h were identified together with AnxA2 and Ahnak (Oh et al., 2013; Figures 1E–G). Smarca3 (SWI/SNF-related, matrix-associated, actin-dependent regulator of chromatin, subfamily A, member 3) is known as a chromatin remodeling factor and is also called helicase-like transcription factor (HLTF; Ding et al., 1999). Supt6h (suppressor of Ty 6 homolog *S. cerevisiae*, also called Spt6) interacts with the C-terminal domain of RNA polymerase II and plays roles in transcription elongation as well as histone 3 modifications (Kato et al., 2013; Cramer, 2019).

The heterotetramer of p11/AnxA2 interacts with a consensus binding motif existing in the N-terminal region of Smarca3 and the C-terminal region of Ahnak (Oh et al., 2013; Figures 1E,G). Formation of the p11/AnxA2/Smarca3 complex increases the DNA-binding affinity of Smarca3 and its localization to the nuclear matrix (Oh et al., 2013), a process which may rely on phospholipid and actin binding properties of AnxA2 (Hayes et al., 2009; Drucker et al., 2014). Chronic SSRIs increase the hippocampal levels of p11 and AnxA2 without changing the level of Smarca3, facilitating the assembly of the p11/AnxA2/Smarca3 complex. SSRI-induced neurogenesis and behavioral responses measured in the NSF and SPT are abolished by constitutive Smarca3 KO (Oh et al., 2013).

SSRI-induced p11 induction was observed in the dentate gyrus, particularly in mossy cells and PV-expressing interneurons, which are also expressing Smarca3 (Oh et al., 2013). Behavioral and neurogenic effects of chronic treatment with the SSRI are abolished by Calcrl-Cre or Drd2-Cre-driven p11 or Smarca3 KO in mossy cells or by inhibition of the p11/AnxA2/Smarca3 heterohexamer with the complex-specific inhibitory peptides (Oh et al., 2020). Chemogenetic activation of mossy cells using Gq-DREADD (Designer Receptors Exclusively Activated by Designer Drugs) is sufficient to elevate the proliferation and survival of neural stem cells. Conversely, acute chemogenetic inhibition of mossy cells using Gi-DREADD impairs behavioral and neurogenic responses to chronic administration of SSRIs (Oh et al., 2020).

Smarca3 KO in PV neurons also abolished the behavioral effects of SSRIs in the FST, TST, and NSF (Umschweif et al., 2021a). Alterations of AMPA receptor signaling and endosomal trafficking were found as downstream events of Smarca3 KO (Umschweif et al., 2021a, b). After chronic treatment with SSRIs, increased levels of p11/Smarca3 complex represses Neurensin-2 gene expression (Umschweif et al., 2021a). Neurensin-2 is localized in endosomal vesicles and potentially involved in vesicular trafficking (Nakanishi et al., 2006; Umschweif et al., 2021b). The behavioral response to SSRIs requires p11/Smarca3-mediated repression of Neurensin-2 expression in PV interneurons (Figure 2D). Additionally, Smarca3 KO or Neurensin-2 overexpression in CCK interneurons (CCK-Cre line), but not PV neurons, in the dentate gyrus causes depressive- and anxiety-like behavioral phenotypes in TST, FST, SPT, cookie test, social interaction test, and OFT (Umschweif et al., 2021b), although CCK-p11 KO did not display baseline depressive-like behavior in the TST and FST (Medrihan et al., 2017). Notably, the accessibility of Neurensin-2 gene (*Nrsn2*) in GABAergic interneurons assessed by ATAC-seq was comparable between WT and Smarca3 KO conditions, making it unclear whether Smarca3 directly or indirectly represses Neurensin-2 gene transcription (Umschweif et al., 2021b). Identification of additional target genes of p11/AnxA2/Smarca3 and p11/AnxA2/Supt6h relevant to the regulation of depressive-like behavior or antidepressant actions would accelerate a full understanding of nuclear p11 pathways (Figures 2D,E).

## p11/AnxA2/Ahnak Pathways in Calcium Signaling and Chronic Stress-Induced Behavioral Responses

The interaction between Ahnak and the p11/AnxA2 heterotetramer has been well characterized (Benaud et al., 2004; Rezvanpour et al., 2011; Oh et al., 2013; Ozorowski et al., 2013). It is also known that Ahnak regulates cardiac L-type voltage-gated calcium channels (VGCC; Haase et al., 1999; Haase, 2007) and L-type Cav1.1-mediated calcium signaling in T cells (Matza et al., 2008, 2009). However, the neuronal function of Ahnak has only recently emerged.

Ahnak is found as a major protein co-precipitated with the p11/AnxA2 complex in a pull-down assay using brain lysates (Jin et al., 2020). Ahnak stabilizes p11 and AnxA2 proteins and scaffolds the L-type pore-forming α1 subunit and the β subunit of VGCC (Figure 1G). Cell surface localization of the α1 subunits and L-type calcium current is significantly reduced in Ahnak KO neurons compared to WT controls, demonstrating L-type VGCC as an effector of the Ahnak/p11/AnxA2 complex (Jin et al., 2020; Figure 2C). Behaviorally, constitutive Ahnak KO mice display a depressive-like phenotype like that of constitutive p11 KO mice in the TST, FST, and SPT (Jin et al., 2020). Forebrain glutamatergic neuron-selective Ahnak KO mice (EMX-Cre crossed with floxed Ahnak) display a depressive-like phenotype. In contrast, PV interneuron-selective Ahnak KO mice (PV-Cre crossed with floxed Ahnak) display an antidepressive-like phenotype.

Differences in Ahnak levels are tightly associated with chronic stress-induced behavioral susceptibility or resilience (Bhatti et al., 2022). Following the chronic social defeat stress (CSDS) paradigm, Ahnak protein in the hippocampus or Ahnak mRNA in PV neurons in the ventral dentate gyrus were reduced in stress-resilient mice but increased in stress-susceptible mice compared to non-defeated control mice. Ahnak deletion selectively in the ventral dentate gyrus or PV neurons resulted in a stress-resilient phenotype measured by the social interaction test and SPT (Bhatti et al., 2022). A decrease in calcium signaling of PV neurons resulting from Ahnak KO may confer stress resilience. In fact, the firing frequency of PV neurons was significantly increased in susceptible compared to non-defeated or resilient mice. Inhibition or activation of PV neurons in the dentate gyrus by chemogenetic tools was capable of conferring resilience or susceptibility to CSDS, respectively, confirming the causal relationship between PV neuronal activity and stress susceptibility or resilience (Bhatti et al., 2022).

Human genetic studies implicate altered function of L-type VGCCs in the pathophysiology of multiple psychiatric disorders including MDD (Green et al., 2010; Liu et al., 2011; Bhat et al., 2012; Cross-Disorder Group of the Psychiatric Genomics Consortium, 2013; Schizophrenia Working Group of the Psychiatric Genomics, 2014; Pinggera et al., 2015). Potential alterations of Ahnak in the brains of MDD patients remain to be investigated. Additionally, Ahnak is highly expressed in endothelial cells in blood vessels (Gentil et al., 2005; Jin et al., 2020). Endothelial cells have been implicated as a target of CSDS, and CSDS-induced dysfunction of the blood-brain-barrier has been suggested as a mechanism underlying stress susceptibility (Menard et al., 2017). Thus, further understanding of the function of Ahnak in other cell types including endothelial cells in the brain will advance our understanding of stress susceptibility and resilience.

## Outstanding Questions

p11 is expressed in many other neuronal cell types and brain regions relevant to mood and anxiety disorders (Milosevic et al., 2017). A study indicates p11 expression in vasopressinergic cells in the paraventricular nucleus where p11 regulates hypothalamic-pituitary-adrenal hyperactivity in a vasopressin V1B receptor-dependent manner (Sousa et al., 2021). Additionally, sympathetic-adrenal-medullary hyperactivity is partially regulated by the loss of p11 in serotonergic neurons of the raphe nuclei (SERT-Cre line bred with floxed p11 line; Sousa et al., 2021). Future studies should identify actual molecular changes of p11 and its pathways in these new cell types with animal models of depression and brain tissues from MDD patients. Importantly, when performing cell-type-specific approaches with animal models for future studies, proper selection of Cre lines and careful interpretations of experimental results are necessary due to potential specificity issues associated with certain cell-type-specific Cre lines (Brindley et al., 2019; Zhao et al., 2019; Muller-Komorowska et al., 2020).

Studies of p11 pathways in non-neuronal cells are another avenue to be explored. p11 and its binding partners are highly expressed in astrocytes, microglia, endothelial cells in blood vessels, ependymal cells in the ventricles, and epithelial cells in the choroid plexus (Gentil et al., 2005; Milosevic et al., 2017; Jin et al., 2020). The deletion of p11 in ependymal cells causes disoriented ependymal planar cell polarity, reduced CSF flow, and depressive-like and anxiety-like behaviors measured by the TST, FST, and NSF (Seo et al., 2021). AnxA2, Smarca3, and Ahnak are all expressed in ependymal cells, and these binding partners together with p11 may regulate calcium signaling and gene expression. Interestingly, previous studies indicate restoration of p11 levels in CINs in the NAc (Alexander et al., 2010; Warner-Schmidt et al., 2012) or glutamatergic neurons in the prelimbic cortex (Seo et al., 2017) in constitutive p11 KO mice sufficiently normalized depressive-like behavior: in the TST and SPT for NAc restoration and in the TST, FST, SPT, and NSF for the prelimbic cortex restoration. It is somewhat puzzling how the restoration of neuronal p11 in one cell type can restore depressive-like behavior driven by the absence of p11 in other neuronal types and non-neuronal cell types.

Finally, alterations of p11 or its binding partners are implicated in pathobiologies relevant to other diseases, including Parkinson’s disease (Zhang et al., 2008; Marongiu et al., 2016; Schintu et al., 2016; Green et al., 2017), drugs of abuse (Arango-Lievano et al., 2014; Thanos et al., 2016), and various cancers (Lokman et al., 2011; Lee et al., 2014; Noye et al., 2018; Bharadwaj et al., 2020; Lu et al., 2020), in which depression is a common comorbid condition. While the human Ahnak gene was initially cloned from tumor cells (Shtivelman and Bishop, 1993) and later characterized as a tumor suppressor (Lee et al., 2014), chemotherapy-induced p11/AnxA2/Supt6h complex is known to facilitate Oct4-mediated gene transcription and thereby is involved in chemotherapy-induced breast cancer stem cell enrichment (Lu et al., 2020). Molecular and cellular studies of p11 and its binding partners using animal models of comorbidities could provide insights into novel mechanisms and innovative therapeutic targets for comorbidities.

## Author Contributions

MC and YK conceptualized and collected references. MC, Y-SO, and YK wrote the manuscript and designed figures. All authors contributed to the article and approved the submitted version.

## Funding

YK was supported by the National Institutes of Health (R01MH121763) and a Seed Grant from the American Epilepsy Society. MC was supported by the National Institutes of Health Institutional National Research Service Award under Award Number T32 GM139776. Y-SO was supported by the National Research Foundation of Korea (NRF) grant funded by the Korea government (MSIT; No. 2021R1A2C1009454), KBRI basic research program through Korea Brain Research Institute funded by Ministry of Science and ICT (22-BR-03-03), and the Bio & Medical Technology Development Program of the National Research Foundation (NRF) funded by the Ministry of Science & ICT (2017M3A9G8084463).
